# Flavonoids’ Dual Benefits in Gastrointestinal Cancer and Diabetes: A Potential Treatment on the Horizon?

**DOI:** 10.3390/cancers14246073

**Published:** 2022-12-09

**Authors:** Raghad Khalid AL-Ishaq, Alena Mazurakova, Peter Kubatka, Dietrich Büsselberg

**Affiliations:** 1 Weill Cornell Medicine-Qatar, Education City, Qatar Foundation, Doha 24144, Qatar; 2 Department of Medical Biology, Jessenius Faculty of Medicine, Comenius University in Bratislava, 036 01 Martin, Slovakia; 3 Biomedical Centre Martin, Jessenius Faculty of Medicine in Martin, Comenius University in Bratislava, Mala Hora 4D, 036 01 Martin, Slovakia

**Keywords:** flavonoid, gut microbiome, diabetes, GI cancer, dual, synergetic, apoptosis

## Abstract

**Simple Summary:**

The consumption of flavonoids positively influences the same (impaired) metabolic pathways in diabetes and gastrointestinal cancers, leading to the hypothesis that flavonoids could exert dual effects on these diseases, as some flavonoids can target the same pathways in both conditions, such as the apoptosis and AMPK pathways. Here, we identified flavonoids with such interactions and discussed their positive effects on both diseases. Nevertheless, more efforts are required to estimate the appropriate flavonoid dosage and flavonoid–flavonoid interactions, and to identify potential side effects. It is also necessary to assess the possibility of combining multiple flavonoids with the currently used treatment.

**Abstract:**

Diabetes and gastrointestinal cancers (GI) are global health conditions with a massive burden on patients’ lives worldwide. The development of both conditions is influenced by several factors, such as diet, genetics, environment, and infection, which shows a potential link between them. Flavonoids are naturally occurring phenolic compounds present in fruits and vegetables. Once ingested, unabsorbed flavonoids reaching the colon undergo enzymatic modification by the gut microbiome to facilitate absorption and produce ring fission products. The metabolized flavonoids exert antidiabetic and anti-GI cancer properties, targeting major impaired pathways such as apoptosis and cellular proliferation in both conditions, suggesting the potentially dual effects of flavonoids on diabetes and GI cancers. This review summarizes the current knowledge on the impact of flavonoids on diabetes and GI cancers in four significant pathways. It also addresses the synergistic effects of selected flavonoids on both conditions. While this is an intriguing approach, more studies are required to better understand the mechanism of how flavonoids can influence the same impaired pathways with different outcomes depending on the disease.

## 1. Introduction

### 1.1. Diabetes

Diabetes is a chronic and complex metabolic disorder with a high prevalence rate worldwide [1]. It is classified into type 1, type 2, and gestational diabetes. These types differ in their diagnostic criteria, genetics, and etiology [2]. Type 2 and gestational diabetes are attributed to unhealthy diet and lifestyle, aging, and urbanization [3]. Diabetes is categorized by hyperglycemia, a continued elevation of blood glucose levels caused either by a defect in insulin secretion or action [4] due to which multiple organs, such as the eyes, heart, and kidneys, become deleterious, leading to organ damage, dysfunction, and, ultimately, organ failure [5,6].

Additionally, complications of diabetes could have profound adverse effects on the social, psychological, and physical health of patients and their families [7]. Symptoms and signs of diabetes include polyuria, polyphagia, polydipsia, blurred vision, and weight loss. Their severity depends on the type and duration of diabetes [8]. Currently, diabetes is treated by administering oral hypoglycemic drugs, antidiabetic drugs, and lifestyle management [9,10]. Further efforts are needed for the development of new therapeutic agents.

### 1.2. Gastrointestinal Cancer

Gastrointestinal cancer (GI) is one of the major causes of death and disability worldwide [11]. The term “GI cancer” describes cancers that affect the digestive system, including, but not limited to, colorectal cancer and gastric cancer [12]. It is a multifactorial disease and can be influenced by environmental and genetic factors such as obesity, diet, smoking, infection, and low socioeconomic status [13,14]. The symptoms of GI cancers depend on their etiology and may include weight loss, fatigue, anorexia, abdominal pain, and dysphagia [15]. GI cancer progression results from the impairment of major metabolic pathways, such as the intrinsic and extrinsic apoptotic pathways, cellular proliferation pathways, and metastatic pathways [16,17]. The field of cancer treatment is evolving continuously, and the applications of precision medicine and combination therapy provide an alternative approach and result in the long-term survival of patients with GI cancers [18]. Despite this, more efforts are required to estimate GI cancers’ underlying mechanisms and develop new therapeutic approaches for prevention and treatment.

### 1.3. Flavonoids Influence Diabetes and GI Cancer

**Figure 1:**
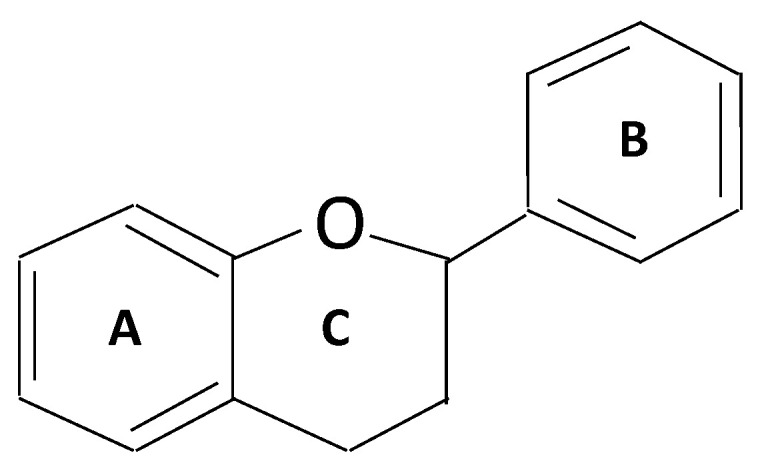
Illustration of the General Structure of Flavonoids.

Flavonoids are secondary metabolites derived mainly from plants, fruits, leaves, and vegetables [19]. They represent a large family of compounds consisting of benzopyran rings with polyphenolic groups at different positions. The structure of flavonoids consists of 15 carbons and two rings (A and B) linked by three carbon chains [20] (Figure 1). Flavonoids are classified into multiple types based on their chemical structure, unsaturated linking chain, and degree of oxidation [21]. Each of these groups possesses biological properties, and some are successfully used in therapeutic approaches for treating diabetes and GI cancer [22]. The administration of flavonoids to target diabetes modulated lipid and carbohydrate metabolisms, improved adipose tissue metabolism, attenuated hyperglycemia, and alleviated oxidative stress [23,24]. Moreover, for targeting GI cancer, flavonoids in berries and green tea reduced cellular proliferation and inflammation, inhibited metastasis, and increased apoptosis [25,26]. Additional efforts are necessary to address the mechanism of flavonoids in treating metabolic disorders such as diabetes and cancer.

### 1.4. Gut Microbiota: The Role in Diabetes and GI Cancer

The human digestive tract harbors several microbial species collectively called the gut microbiome [27]. The human gut microbiome influences health and disease status through its involvement in human nutrition, immunity, and physiology [28]. Recently, we reported the effect of gut microbiome enzymatic modifications on flavonoid metabolism and how these modifications affect the observed biological properties of flavonoids [29].

**Figure 2:**
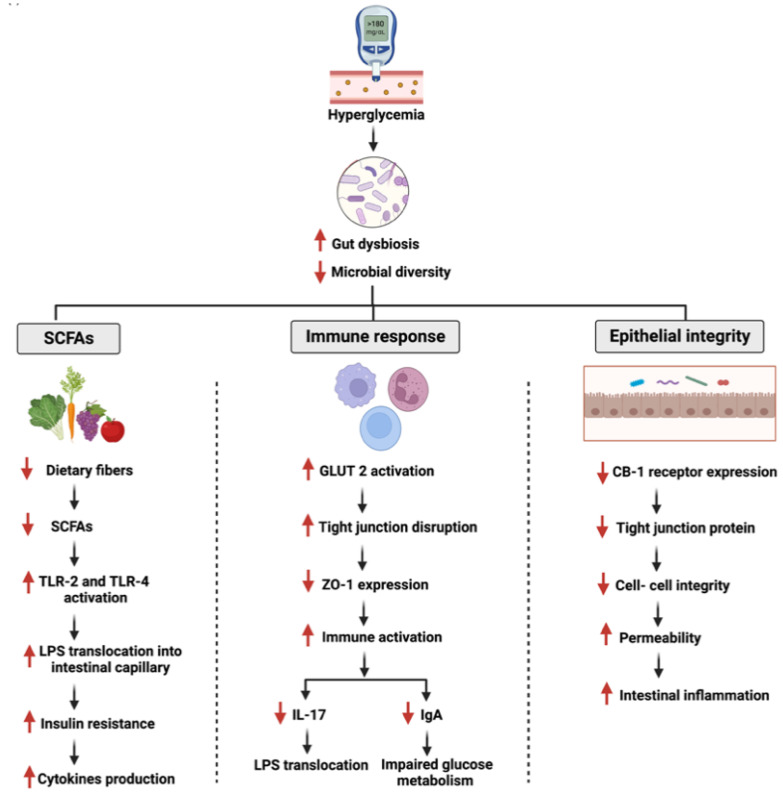
Illustration of the effects of diabetes on gut microbiome. The figure is divided into three sections highlighting three impaired pathways: short chain fatty acid production, immune response, and epithelial integrity. SCFA: Short Chain Fatty Acids. Created with BioRender.com.

**Figure 3:**
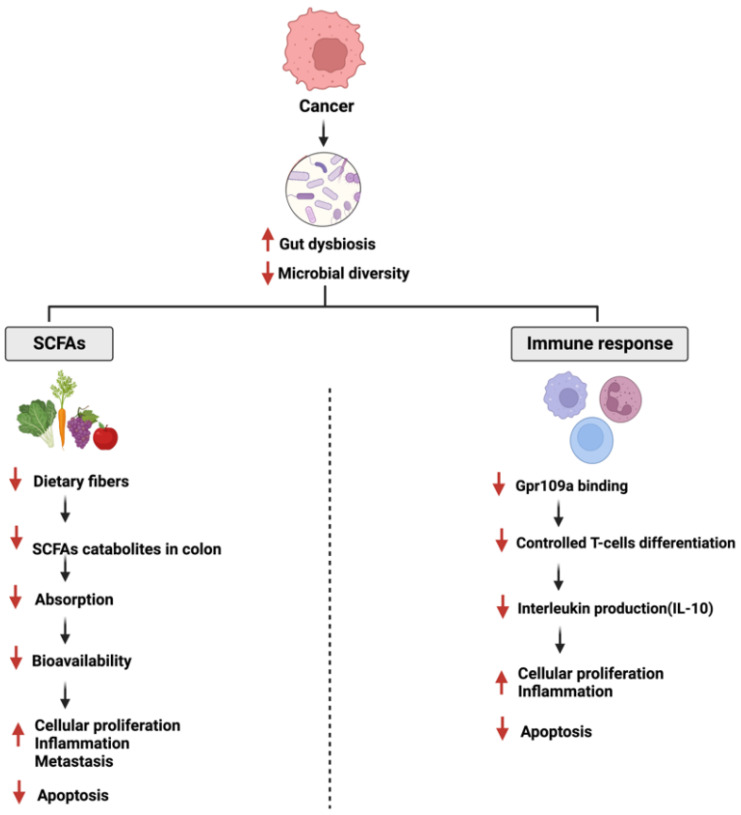
Illustration of the effects of cancer on gut microbiome. The figure is divided into two sections highlighting two impaired pathways: short chain fatty acid production and immune response. SCFA: Short Chain Fatty Acids. Created with BioRender.com.

Human gut microbiome diversity disruption is linked to pathological conditions such as inflammatory bowel disease (IBD), obesity, diabetes, and cancers [30,31]. In two population-based studies, the diversity and richness of the gut microbiome were associated directly with the development of type 2 diabetes and insulin resistance [32]. Furthermore, the gut microbiome’s impact on cancer is not only limited to specific pathways such as apoptosis and cellular proliferation, but also enhances the efficacy and reduces the resistance of chemotherapeutic drugs [33]. Figure 2 and Figure 3 summarize the overall interactions between the gut microbiome and specific pathways during diabetes and GI cancer.

In our previous work, we identified the role of different flavonoids on diabetes and GI cancer separately [34,35]. In this review, we evaluate and analyze published studies to report and estimate the possible dual role of flavonoids in targeting diabetes and GI cancers. The literature supports a potential association between both conditions, as hyperglycemia level influences the development of diabetes and GI cancers. Furthermore, we assess the impact of this interaction on specific and common pathways in both metabolic conditions. Finally, we identify gaps in the current research.

## 2. Search Strategy and Selection Criteria

Medline, Scopus, and PubMed were searched for manuscripts published from 2000 to 2022 using the search terms “flavonoids”, “microbiota”, “flavonoids AND diabetes”, “flavonoids AND cancer”, “flavonoids AND GI cancer”, “microbiome AND GI cancer”, “gut microbiota enzymes”, microbiome AND diabetes”, and “flavonoids AND GI cancer AND diabetes”. In this article, we selected 121 articles and analyzed them in detail. Eligible studies included in vivo, in vitro, and clinical trial papers addressing the influence of flavonoids on both conditions and the possible underlying dual effects. Duplicates and studies with other flavonoid metabolism mechanisms were excluded.

## 3. Role of Flavonoids on Diabetic and GI Cancer Pathways

**Figure 4:**
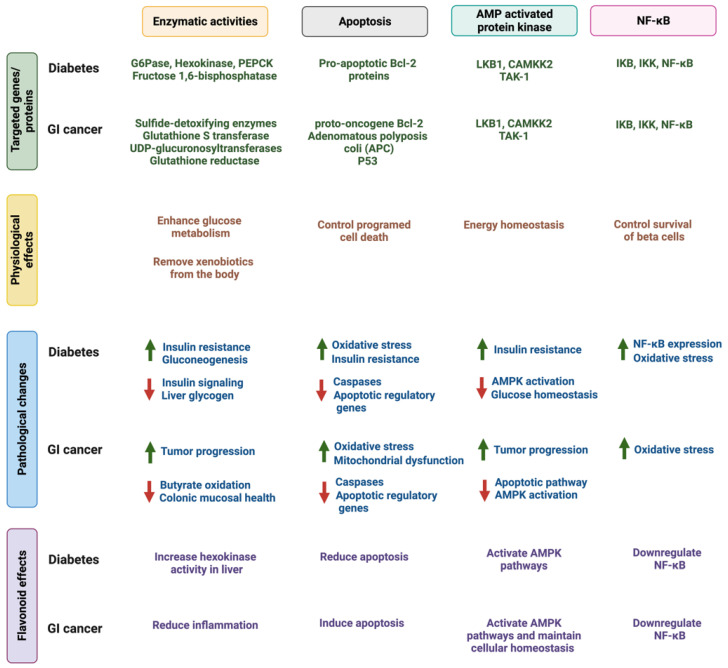
The four commonly impaired pathways in diabetes and GI cancer. The figure highlights the main genetic target, pathological changes during the disease, and the reported influence of flavonoids on these changes. Created with BioRender.com.

As discussed in the previous section, flavonoids exhibit diverse biological properties that target diabetes and GI cancers. In the following paragraphs, we will discuss the influence of specific flavonoids on major impaired pathways in diabetes and GI cancers such as apoptosis, cellular proliferation, enzymatic modifications, and AMPK pathways. Figure 4 highlights an overview and summarizes the impaired pathways in both metabolic conditions and the effects of flavonoids in targeting them.

### 3.1. Flavonoids and Apoptosis in Diabetes and GI Cancers

Cellular apoptosis is programmed cell death that involves numerous regulatory genes [36]. These regulatory checkpoints are critical to maintaining a homeostatic balance between the newly formed and damaged cells [37]. Loss of this balance may lead to the development of diseases such as diabetes and/or GI cancers [38]. Flavonoids restore the imbalance in these conditions [39].

In diabetes, long-term hyperglycemia in patients may contribute to the accumulation of reactive oxygen species (ROS), which results in endothelial cellular stress [40]. If unresolved, it may lead to apoptosis of microvascular endothelial cells, which, in turn, results in diabetic complications such as diabetic cardiomyopathy and diabetic nephrosis [41]. However, baicalin, a flavone glycoside in tea, fruits, and vegetables, reduces apoptosis in a rat model of diabetes mellitus, thus supporting the neuroprotective efficacy of baicalein against diabetes-associated cognitive deficits [42]. Moreover, a study that stimulated diabetic mice cells with 60.0 M of glucose to induce oxidative stress showed that cells treated with baicalin had significantly alleviated oxidative stress and apoptosis through the regulation of PERK/Nrf2 pathway [43]. Additionally, hesperidin, a glycosidic flavonoid abundant in citrus fruit, also reduces oxidative stress [44]. Normal human hepatocytes stimulated with 33 mM of glucose for 24 h and then treated with hesperidin had improved oxidative stress levels and increased cell viability compared to the untreated cells. These findings demonstrated that hesperidin treatment might reduce apoptosis by regulating miR- 149 expression, a critical apoptotic regulator [45]. Similarly, rutin also alleviated diabetic complications through apoptosis [46]. Myoplast cells stimulated with different glucose concentrations and then treated with rutin showed an improvement in cardiomyocyte injury through the direct inhibition of apoptosis and endoplasmic reticulum stress [47].

**Figure 5:**
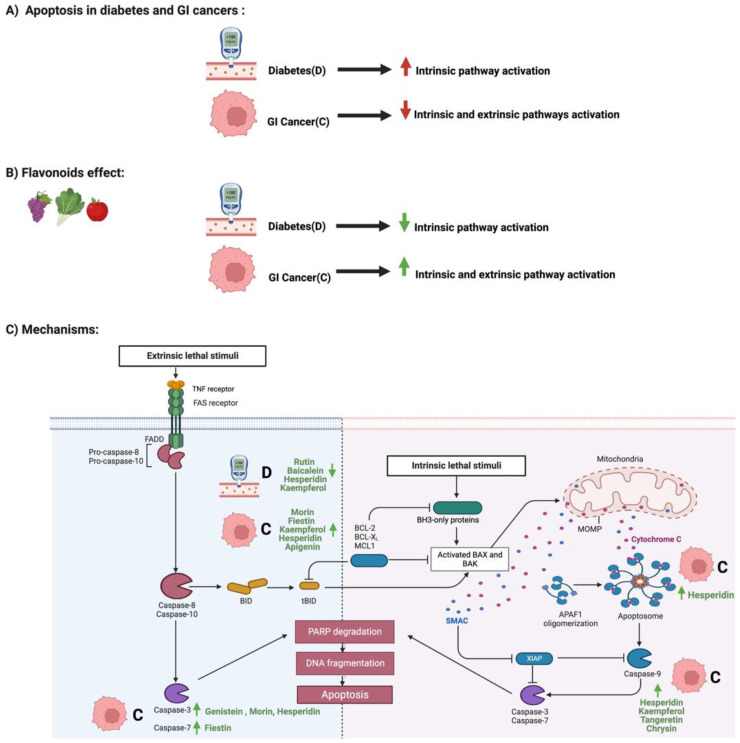
Illustrations of the influence of specific flavonoids on apoptotic pathways in diabetes and GI cancers. The figure highlights the pathological changes in apoptosis during both conditions, the flavonoids’ effect, and the mechanisms through which the flavonoids target the pathway. Created with BioRender.com.

The reduction of apoptotic regulation allows cancerous cells to survive and progress [48]. Flavonoids such as apigenin, a plant flavone, induce cell cycle arrest, disruption, and apoptosis with low toxicity and mutagenicity rates [49]. Additionally, 200 umol/L of genistein for 48 h affected both Notch 1 and epithelial-mesenchymal transition (EMT) pathways and significantly induced apoptosis in colorectal cancer cells [50]. Moreover, the administration of 50 uM of Kaempferol also decreased the Bcl-2 level and increased the cleaved caspase 9 expression, thus inducing apoptosis in gastric cancer cells [51]. Interestingly, the same apoptotic pathway may be targeted by different flavonoid types such as chrysin, morin, and hesperidin. All three flavonoids induced apoptosis separately through the modulation of caspase 3, 9, and BAX expression in colorectal cancer or gastric cancer cells [52,53,54]. Moreover, cotreatment of apigenin and 5-flurouracil increased ROS and mitochondrial membrane potential in colorectal cancer cells, supporting flavonoids’ potential role as a therapeutic agent [55]. Figure 5 summarizes the influence of specific flavonoids on the apoptotic pathways in diabetes and GI cancers.

### 3.2. Flavonoids and NF-κB Pathway in Diabetes and GI Cancers

Nuclear factor kappa B (NF-κB) transcription factors are critical regulators of the inflammatory and immune responses against infections and injuries [56]. NF-κB dysregulation may lead to the development of cancers, chronic inflammation, and insulin resistance [57]. The activation of NF-κB by oxidative stress in diabetic patients due to the high glucose level plays a crucial role in the vascular complications of diabetes [58]. Additionally, persistent hyperglycemia in diabetes triggers the expression of chemokines, cytokines, and cell adhesion molecules by activating NF-κB expression [59]. Furthermore, activation of the NF-κB pathway correlates with GI cancers’ tumor size, migration, and invasion through the modulation of inflammatory markers, just as in the pathogenesis of GI cancers [60]. Targeting the NF-κB pathway with flavonoids is reported to be a potential therapeutic target for diabetes and GI cancers [61,62].

Different flavonoids exert antidiabetic properties. Baicalin, a bioactive polyphenolic flavonoid, administered to diabetic mice significantly reduced glucose levels and inhibited insulin levels [63]. Additionally, the administered baicalin attenuated steatosis in diabetic mice’s hepatic tissues through the suppression of inflammatory cytokines regulated by the NF-κB pathway. Treatment with 20 mg/kg of apigenin also reduced NF-κB and TNF-a expression and inhibited mouse inflammations [64]. Moreover, luteolin significantly reduced oxidative stress by inhibiting the NF-κB and activating the nuclear factor-erythroid 2-related factor 2 signaling pathway [65]. Interestingly, as reported with quercetin, flavonoids inhibit NF-κB expression and target vascular complications in diabetes [66]. Daily administration of quercetin in rats for six weeks reduced NF-κB expression and protects against diabetic-induced exaggerated vasoconstriction.

**Figure 6:**
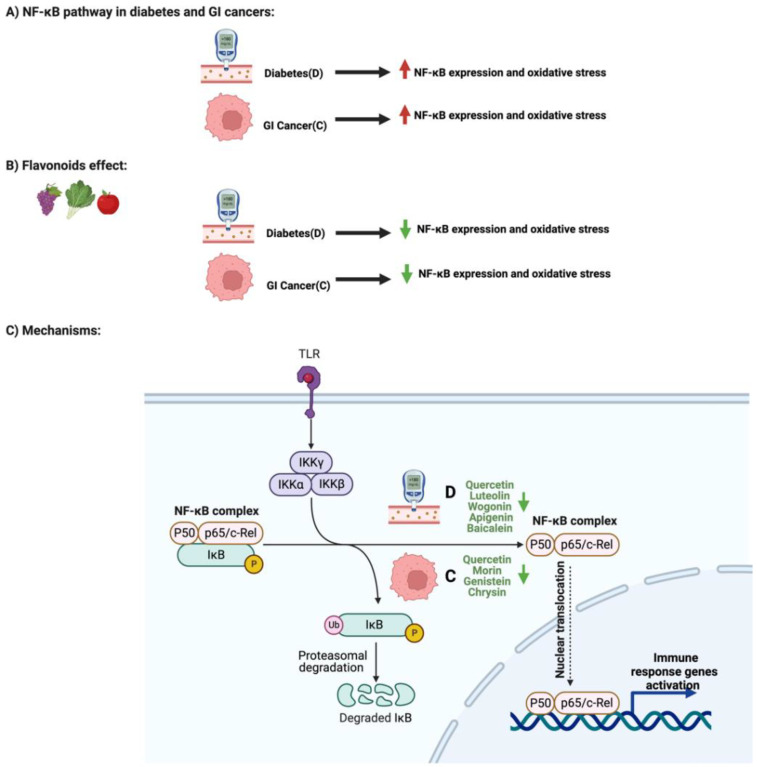
Summary of the influence of specific flavonoids on NF-κB pathways in diabetes and GI cancers. The figure highlights the pathological changes in the pathway with both conditions, the flavonoids’ effect, and the mechanisms through which the flavonoids target the pathway. Created with BioRender.com.

Flavonoids also play a significant role in targeting the NF-κB pathway through different mechanisms in gastric cancer (GC). Chrysin, for example, significantly suppressed NF-κB and Egr-1 expression in gastric cancer cells by inhibiting endogenous recepteur d’órigine nantais (RON) expression and activity, a critical receptor for cancer cell invasion [67]. Additionally, treating colorectal cancer cells with morin suppressed the expression of NF-κB and the production of inflammatory cytokines such IL-8 and IL-6 [68]. Furthermore, genistein administration in gastric cells targeted and significantly reduced the expression of Cyclooxygenase-2 (COX-2), an isoenzyme critical in cancer progression, by targeting and inhibiting its regulator, NF-κB [69]. Quercetin also downregulated the NF-κB pathway, influencing the invasion, migration, and metastasis of gastric cancer cells [70]. Figure 6 highlights the influence of specific flavonoids on the NF-κB pathway and expression in diabetes and GI cancers.

### 3.3. Flavonoids and AMPK Pathway in Diabetes and GI Cancers

AMPK-activated protein kinase is a highly conserved protein kinase and a central regulator of cellular energy and homeostasis in eukaryotes [71]. It exists in the cell as an obligate heterodimer with a catalytic subunit and two regulatory subunits [72]. Several cellular stressors, such as hypoxia, can activate the AMPK pathway [73]. Impaired regulations of the AMPK pathway play an essential role in developing metabolic conditions such as diabetes, leading to insulin resistance and cancers and tumor progression [74,75]. However, flavonoids could positively influence and regulate AMPK pathways in diabetes and GI cancers.

Three main flavonoids regulate the AMPK pathway in diabetes: quercetin, daidzein, and Kaempferol. Skeletal muscle cells treated with quercetin induced glucose uptake in the cells, changed the mitochondrial membrane potential, and increased intracellular calcium concentration [76]. The observed antidiabetic effects were modulated mainly through the regulation of the AMPK pathway and GLUT4 expression and were similar to the mechanisms of the well-known antidiabetic drug metformin [76]. Additionally, diabetic mice and myotubes cells treated with daidzein showed that this flavonoid promoted glucose uptake through the phosphorylation of AMPK and the translocation of GLUT4 expression [77]. Moreover, the administration of Kaempferol to pancreatic cells upregulated the phosphorylation of the AMPK pathway. When an inhibitor was used, it reduced the pancreatic cells’ survival rate suggesting a potential cytoprotective effect of Kaempferol on diabetes [78].

**Figure 7:**
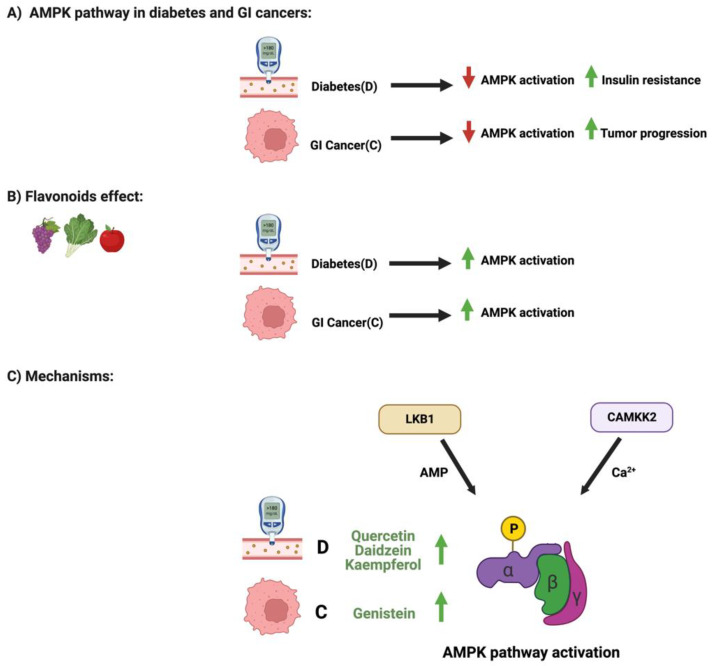
Schematic illustration of the influence of specific flavonoids on AMPK pathways in diabetes and GI cancers. The figure highlights the pathological changes in the pathway with both conditions, the flavonoids’ effect, and the mechanisms through which the flavonoids target the pathway. Created with BioRender.com.

On the other hand, colorectal cancer cells treated with a soy-derived phytochemical, genistein, demonstrated activated AMPK expression [79]. Additionally, combination therapy of genistein and 5-flurouracil abolished cellular growth, induced cellular apoptosis, and activated AMPK phosphorylation, exerting a potential chemotherapeutic approach against colorectal cancers. Figure 7 illustrates the influence of specific flavonoids on the AMPK pathway and expression in diabetes and GI cancers.

### 3.4. Flavonoids and Enzymatic Activities in Diabetes and GI Cancers

Enzymes are critical modulators in the body, aiding in regulating the metabolic process and chemical reactions [80]. Their influence on these reactions depends on the target tissue and regulatory mechanisms [81]. An imbalance in the regulation or the expression of specific enzymes, such as glucose 6 phosphatase or glutathione reductase, may lead to diabetes and cancers, respectively [82,83].

In diabetes, the level of hepatic enzymes such as glucose 6 phosphatase and phosphoenolpyruvate carboxykinase increases, while the level of hexokinase decreases. These enzymes are critical in maintaining blood glucose regulation in the body [84]. Flavonoids such as genistein, diosmin, morin, rutin, and baicalin improve the activities of these enzymes in diabetic models. The administration of genistein (0.02%) in mice reduced glucose 6 phosphatase and phosphoenolpyruvate carboxykinase activities in the liver compared to the control [85]. Additionally, oral administration of diosmin (100 mg/kg) significantly decreased glucose 6 phosphatase activities and increased hexokinase in diabetic-induced rats [86]. Moreover, rutin administration for 30 days to diabetic rats significantly ameliorated hyperglycemia, decreased glucose 6 phosphatase activities, and increased hexokinase activities [87]. Furthermore, glucose 6 phosphatase and hexokinase activities were restored after the oral administration of morin for 30 days in diabetic rats [88]. These data support the potential role of flavonoids in influencing the hepatic enzymatic activities in the liver, which may be applicable for diabetic management.

**Figure 8:**
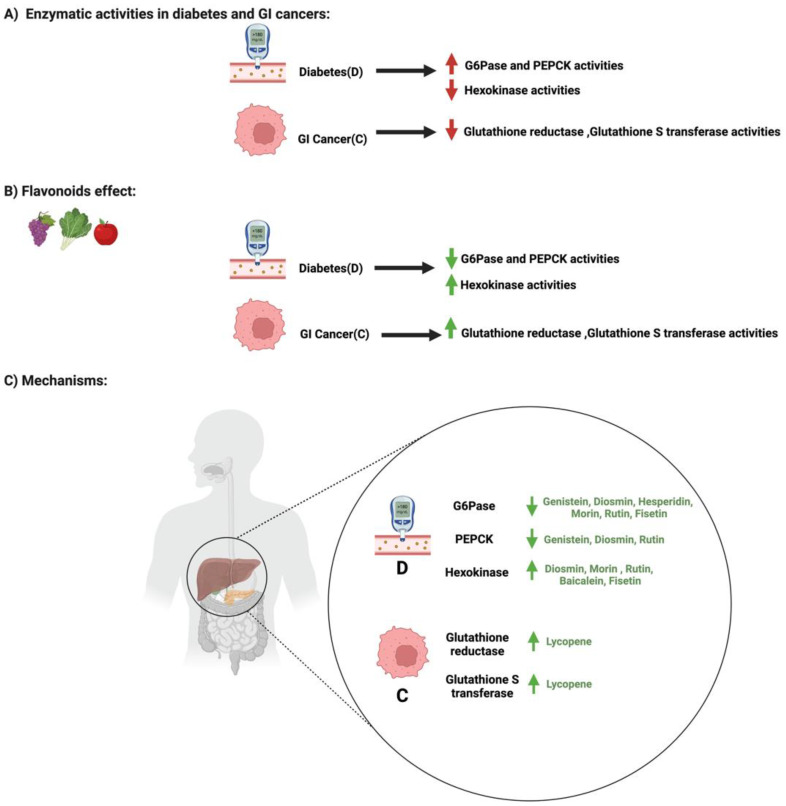
The influence of specific flavonoids on the enzymatic modification pathways in diabetes and GI cancers. The figure highlights the pathological changes in the pathway with both conditions, the flavonoids’ effect, and the mechanisms through which the flavonoids target the pathway. Created with BioRender.com.

In contrast, in GI cancers, enzymes such as glutathione reductase and glutathione S transferase are critical in regulating glutathione utilization, a cellular regulator for the redox state [89]. An imbalance in the utilization of this intracellular antioxidant may impair biological pathways such as apoptosis and cellular proliferation [90]. Limited studies reporting the effect of flavonoids on glutathione utilization enzymes in GI cancers are available. Lycopene administration (2.5 mg/kg) suppressed gastric cancer by increasing glutathione reductase and glutathione S transferase activities [91]. More studies are required to confirm this observation and support the potential role of flavonoids in GI cancer pathogenesis. Figure 8 shows the influence of specific flavonoids on enzymatic activities and expression in diabetes and GI cancers. Table 1 and Table 2 summarize the main findings from the reported studies.

## 4. Possible Dual Effects of Flavonoids on Diabetes and GI Cancer

As illustrated in the tables above, we identified three main flavonoids targeting metabolic pathways that are impaired in diabetes and GI cancers. These flavonoids are hesperidin, Kaempferol, and quercetin, and they target apoptosis and the NF-κB pathways This could suggest possible dual effects of flavonoids on diabetes and GI cancer. In male diabetic rats, the administration of hesperidin (100 mg/kg) for four weeks significantly reduced blood glucose and increased insulin levels [104]. Additionally, and more importantly, hesperidin effectively regulated the expression of apoptotic proteins, as shown by the upregulation of the antiapoptotic Bcl-xl level and the downregulation of the pro-apoptotic Bax level [104].

Moreover, in three gastric cancer cell lines, the treatment of hesperidin also targeted apoptotic regulatory proteins but with a different biological effect. The administered hesperidin upregulated the pro-apoptotic Bax level and downregulated the antiapoptotic Bcl-2 levels [54]. Despite the differences observed in both studies (ranging from the targeted disease, the model used, the concentration of the hesperidin, the administration route, and the duration of the treatment), hesperidin affected apoptotic proteins and restored their physiological effects in both studies, but in an opposite manner, resulting in reduction in diabetes and induction in GI cancers. Moreover, Kaempferol also targeted the apoptotic pathway in both conditions. In diabetes, Kaempferol significantly improved the expression of antiapoptotic proteins such as Bcl-2, inhibited apoptosis, and reduced caspase 3 activities in INS-1E beta cells [105]. Kaempferol also targeted apoptosis in colorectal cancer cells by blocking ROS production [106]. Although these results may support the potential efficacy of hesperidin and Kaempferol to modulate apoptosis in different metabolic conditions, more efforts are required to test both conditions in alignment using a standardized technique.

As discussed in the previous section, quercetin can affect NF-κB pathways in diabetes and GI cancers. Diabetic mice treated with orally administered quercetin for two weeks showed a suppressed inflammatory response indicated by reduced NF-κB expression [96]. In gastric cancer cells, quercetin also inhibited the dose-dependent NF-κB pathway [107]. However, limited data are available in the literature to support the hypothesis that quercetin may have the same biological effect on the two conditions: diabetes and GI cancers.

Analyzing the available data on three flavonoids, the possible dual effect depends on the targeted pathway, the model used, and the flavonoid dosage, which needs standardization. Additionally, studies evaluating the impact of these flavonoids in combination with the currently used treatment are lacking in the literature; assessing the potential of dual effects of other flavonoids on diabetes and GI cancers is also highly required.

## 5. Discussion

### 5.1. Targeting Multiple Pathways with Multiple Flavonoids That Act Synergistically?

As mentioned in the previous sections and the figures, the same flavonoid can trigger and improve diabetes and/or cancer-related pathways, as seen with baicalin and genistein, respectively. Baicalin, for example, exerts its antidiabetic effects by targeting three out of four discussed pathways: apoptosis, NF-κB, and enzymatic modification. Genistein, on the other hand, triggers apoptosis, NF-κB, and AMPK pathways in cancer. Therefore, can the positive effects of flavonoids on physiological pathways be further enhanced when different flavonoids are combined to complement and trigger additional pathways? For instance, with baicalin targeting three diabetic-impaired pathways, combining baicalin with one of the flavonoids that target the AMPK pathway in diabetes (Figure 7) might potentially initiate a positive effect in all four of the discussed pathways due to synergistic effects of both phytochemicals. This hypothesized approach may prevent the over-activation of the same pathway and reduce possible side effects while achieving the antidiabetic effects of these flavonoids. The same approach may also be used with genistein in GI cancer. So far, these thoughts are theoretical and only based on observation from the data cited above. In vitro or in vivo studies are still missing, and extensive studies are required to prove whether they are effective in an expected way. This hypothesis may be tested by first identifying the pathways targeted by the flavonoid in each metabolic condition, then identifying flavonoids that complement each other. Furthermore, estimating the appropriate concentration of each flavonoid is critical to ensure safety and prevent cytotoxicity. Recognizing the potential binding sites of each flavonoid and their bioavailability is essential to ensure their positive effects. Moreover, observing possible side effects from this combination with the currently used treatment is necessary, as the aim is not to replace the available therapeutic tools, but to complement them.

### 5.2. Gut Microbiome and Flavonoids’ Effects

The relationship between the gut microbiome and flavonoids may be described as a bidirectional interaction. On the one hand, the consumed flavonoids act as a regulator of intestinal hemostasis by either enhancing or suppressing the growth of specific bacterial species. On the other hand, the gut microbiome contributes to the pharmacokinetics of ingested dietary flavonoids to produce aglycones and, subsequently, the ring fission products which facilitate absorption [108,109,110]. It is hypothesized that these enzymatically modified gut microbiome products are responsible for the positive physiological effects of flavonoids observed in diabetes and GI cancer (Figure 2 and Figure 3). This may provide a potential therapeutic target for both metabolic conditions. Patients with type 2 diabetes exhibit gut microbiome dysbiosis characterized by a reduction in butyrate-producing bacterial species [111]. Additionally, the gut microbiome regulates the levels of short-chain fatty acids and lipopolysaccharides, which are critical for developing diabetes [112]. Furthermore, patients with type 2 diabetes exert a low-grade systemic inflammation mediated by the impaired gut barrier [113].

On the other hand, gut microbial species such as *Streptococcus bovis*, *Fusobacterium nucleatum,* and *Enterococcus faecalis* mediate inflammatory response and tumor progression in colorectal cancer [114,115]. The data above show that flavonoids and their metabolites could reduce or restore the observed pathological effects in diabetes and GI cancers. Despite this, more efforts are required to properly study the gut microbiome, including a suitable model and flavonoid interactions. More studies are required to evaluate whether the positive/dual effects observed are due to the flavonoids, their metabolites, or both. In addition, more efforts are needed to address the metabolites from the gut microbiome and the liver, and especially to discuss and understand the substances metabolized in the liver [116]. The development of more omics tools that help analyze data is necessary [117].

### 5.3. Challenges with Studying the Field

Although flavonoids reduce the pathogenesis of diabetes and GI cancers, further efforts are required to address the main challenges of their utilization in managing cancer and diabetes. First, the recommended dosage of flavonoids for each metabolic condition must be established. The literature shows variations in the estimated dosage for most flavonoids tested based on the population, ethnicity, gender, and geographical locations [118,119,120]. The observed variations may be due to the solubility and molecular weight of the flavonoids used [121]. Second, newly developed tools and techniques are required to overcome flavonoids’ relatively low bioavailability rate.

Additionally, a better understanding of flavonoids’ cellular permeability, excretion, and metabolic alternation is needed to enhance their bioavailability [122]. Third, the potential side effects of using multiple flavonoids to target various metabolic pathways or using flavonoids in combination with currently used drugs as a treatment method must be estimated. This will help to minimize the risk of negative flavonoid–drug interactions. Fourth, the field is in great need of standardization starting from the model used, flavonoid extraction and purification technique, administration route, and duration of the treatment. By overcoming these challenges, using flavonoids as a potential therapeutic target may be implemented with high effectiveness and safety levels.

### 5.4. GI Cancers and Diabetes: Their Relationship?

The prevalence of diabetes mellitus among patients with GI cancers varies depending on the tumor type. For example, the highest rate of diabetes mellitus was seen in patients with colon cancer (15.5%), rectal cancer (15.3%), and stomach cancer (14%) [123]. On the other hand, diabetes in patients can promote the progression, development, and metastasis of gastric cancers [124]. The observed association may be due to the shared risk factors between both conditions such as diet, smoking, infection, medication, obesity, and hyperglycemia [125]. More data are still needed to identify which condition comes first and whether we can predict the occurrence of one following the other. Despite this, the available data suggest a possible association between both conditions and how they may be targeted using flavonoids as a novel therapeutic agent, which might help improve the quality of the patients’ lives and reduce the burden of both conditions.

## 6. Conclusions

Flavonoids are naturally occurring products found abundantly in fruits and vegetables. Upon ingestion, flavonoids are further metabolized by the gut microbiome into aglycones that exert antidiabetic and anti-GI cancer effects. Based on the current evidence, flavonoids target impaired pathways such as apoptosis and NF-κB in both conditions [126,127]. Despite this, the mechanisms by which flavonoids target the same pathway with different biological effects depending on the metabolic conditions need further research. Only limited evidence from the literature supports a potential dual effect of flavonoids.

Moreover, a combination of several flavonoids (affecting the pathology synergistically or additively) represents a promising strategy to be further evaluated to target impaired pathways. Still, more efforts are required for protocol standardization to ensure safety and effectiveness. Similarly, vitexin, an apigenin flavone glucoside, exerts beneficial effects in high glucose-induced endothelial cells via inhibition of apoptosis and oxidative stress, thus suggesting the potential effectiveness of vitexin in cardiovascular complications of diabetes. Specifically, vitexin decreased apoptosis via the disruption of Wnt/β-catenin and Nrf2, decreased ROS and malondialdehyde content, and increased superoxide dismutase activity in HG-mediated HUVECs [128]. In addition to isolated flavonoid compounds, extracts rich in flavonoids were also reported to influence metabolic pathways, such as *Oldenlandia diffusa*, which induced apoptosis via ROS accumulation, mitochondrial membrane potential loss, and activation of mitochondrial pathway in gastric cancer in vitro and in vivo [129]. In this regard, a lack of understanding of flavonoids’ complex mechanisms of action, non-specific selectivity, and low bioavailability in organisms markedly impedes their pharmacological administration in clinical practice. Mentioned obstacles could be overcome using progressive methodological approaches in medicine, i.e., designing nano-engineering systems to achieve the delivery of flavonoids into targeted organs and tissues. Such techniques could improve flavonoids’ efficacy and pharmacokinetic properties in diabetic cancer patients.

Our paper provides a theoretical base for advancing highly effective and low-toxicity flavonoid-based pharmaceutical molecules or their mixtures to target complex signaling pathways associated with diabetes and cancer concurrently. However, only precisely designed and well-controlled clinical evaluations of the context of the antidiabetic and anti-cancer effects of bioactive flavonoids could establish the role of these polyphenolic compounds as beneficial agents in diabetic and GI cancer patients.

## Figures and Tables

**Table 1:** Representative Flavonoids Mechanisms and their Underlying Antidiabetic Effects.

Targeted Pathway	Name of Flavonoid	Flavonoid Subclass	Metabolites Produced by Gut Microbiota	Disease(s)	Influence of Flavonoids on Disease	Methods of Testing	Model Used		References
							In vivo	In vitro	
Enzymatic Modification	Rutin	Flavonol	Quercetin-3-O-glucoside Quercetin	Diabetes	* Enhanced the activity of liver hexokinase * Decreased the activities of glucose-6-phosphatase (G6Pase)	Oral glucose tolerance test (OGTT) Histology RNA extraction Reverse transcription PCR	* White male albino rats	* Tissues from the albino rats	[87]
	Fisetin	Flavonol	No available data	Diabetes	* Inhibited hepatic gluconeogenesis * Decreased the activities of glucose-6-phosphatase (G6Pase) * Decreased the activities of Phosphoenolpyruvate carboxykinase (PEPCK)	Western blot analysis ELISA RNA extraction Reverse transcription PCR	* Male albino Wistar rats	* Liver tissues	[92]
	Morin	Flavonol	Morin glucuronides Morin sulfates	Diabetes	* Significantly reduced blood glucose * Morin treatment significantly reduced the G6Pase activities * Increased insulin levels and hexokinase activities	Intraperitoneal glucose tolerance test (IPGTT) Histopathological examination Immunohistochemical staining	* Male Albino Wistar rats	* Pancreatic tissue	[88]
	Hesperidin	Flavanones	Hesperetin	Diabetes	* Increased insulin levels * Hesperidin treatment normalized the G6Pase activities	Blood glucose meter Liquid chromatography ELISA	* Male Wistar rats		[93]
	Genistein	Isoflavones	Dihydrogenistein 6-hydroxy-O-desmethylangolensin 2-(4-hydroxyphenyl) propionic acid	Diabetes	* Decreased the activities of glucose-6-phosphatase (G6Pase) * Decreased the activities of Phosphoenolpyruvate carboxykinase (PEPCK)	Intraperitoneal glucose tolerance test (IPGTT) Radioimmunoassay Immunohistochemistry	* Female NOD mice	* Pancreatic tissue *Liver tissues	[85]
	Baicalein	Flavones	Baicalein	Diabetes	* Hepatic hexokinase was significantly increased after the administration of 25, 50 or 10 mg/kg of baicalein	Histopathological examination ELISA	* Male Wistar rats	* Pancreatic tissue * Liver tissues	[94]
	Diosmin	Flavones	Diosmetin	Diabetes	* Decreased the activities of glucose-6-phosphatase (G6Pase) * Decreased the activities of Phosphoenolpyruvate carboxykinase (PEPCK) * Enhanced hexokinase activities	ELISA	* Male albino Wistar		[86]
NF-κB Pathway	Baicalein	Flavones	Baicalein	Diabetes	* Baicalein remarkably supressed the inflammatory cascade such as Myd88 and NF-κB thus inhibiting the production of interleukin (IL)-1β and IL-6	Reverse transcription PCR Flow cytometry Western blot analysis	* C57BL/KsJ-db/db mice	* MPC-5 cells * HepG2 cells	[63]
	Luteolin	Flavones	No available data	Diabetes	* Luteolin significantly inhibited the activation of nuclear factor-kappa B (NF-κB) pathway	Reverse transcription PCR Tissue histology Western blot analysis Cell viability	* Male C57BL/6 mice	* H9C2 cells	[65]
	Apigenin	Flavones	Apigenin 3-(4-hydroxyphenyl) propionic acid	Diabetes	* The intragastric administration of apigenin-SLNP supressed the expression of nuclear factor-kappa B (NF-κB) pathway * Protective effect of apigenin against diabetic nephropathy	Superoxide Dismutase (SOD) Reverse transcription PCR Histopathological Changes Western blot analysis	* Rats	* Kidney tissue	[64]
	Wogonin	Flavones	Wogonin	Diabetes	* The intragastric administration of wogonin (10, 20, and 40 mg/kg) for 13 days attenuated histological damages and regulated inflammation through nuclear factor-kappa B (NF-κB) pathway	Immunohistochemistry Assay RNA Isolation Reverse transcription PCR ELISA	* C57/BL male mice	* HK-2 cells	[95]
	Quercetin	Flavonol	3,4-dihydroxyphenylacetic acid 3-(3-hydroxyphenyl) propionic acid 3,4-dihydroxybenzoic 4-hydroxybenzoic acid	Diabetes	* Daily administration of quercetin for six weeks showed inhibitory effects on inflammatory NF-κB signaling pathway	Immunohistochemistry Histopathological Examination	* Male albino rats		[66]
AMPK Pathway	Kaempferol	Flavonol	Kaempferol -3-O-glucoside p-coumaric acid kaempferol 3-(4 hydroxyphenyl) propionic acid 3-phenylpropionic acid	Diabetes	* Kaempferol illustrated cytoprotective effects during diabetes through the upregulation of AMPK phosphorylation * Kaempferol administration enhanced the metabolism of lipid through the activation of AMPK pathway	Cytotoxicity assays DNA ladder assay Electron microscopy Flow cytometry Reverse transcription PCR	* Female Sprague-Dawley rats	* RIN-5F cells	[78]
	Quercetin	Flavonol	3,4-dihydroxyphenylacetic acid 3-(3-hydroxyphenyl) propionic acid 3,4-dihydroxybenzoic 4-hydroxybenzoic acid	Diabetes	* Quercetin administration enhanced the AMPK activation and lipid metabolism	PCR Western blot analysis Immunofluorescence Assay	* Male Wistar rats	* L6 myoblast	[96]
	Daidzein	Isoflavones	Dihydrodaidzein O-desmethylangolensin S- equol	Diabetes	* Administration of daidzein markedly improved the AMPK phosphorylation	Protein isolation Western blot analysis PCR ELISA	* Male KK-Ay/Ta Jcl mice * C57BL/6J Jcl mice	* L6 myoblasts	[77,97]
Apoptosis	Baicalein	Flavones	Baicalein	Diabetes	* The administration of baicalein significantly reduced oxidative stress and apoptosis in cells stimulated with glucose	Fluorescence microscopy Flow cytometry Cell viability assays Immunoblotting Quantitative PCR	* Male Wistar rats	* HL-7702 cells	[42,43]
	Rutin	Flavonol	Quercetin-3-O-glucoside Quercetin	Diabetes	* Rutin administration to myoplastic cells indicated that rutin can, in a dose-dependent manner, reduce the level of endoplasmic reticulum stress and apoptosis	TUNEL assay Cell counting kit-8 assay Western blot analysis		* H9C2 myoblast cells	[47]
	Hesperidin	Flavanones	Hesperetin	Diabetes	* Induced hyperglycemic mice administered with hesperidin downregulated antiapoptotic proteins such as Bcl-2 and Bcl-XL and upregulated the pro-apoptotic proteins	Western blot analysis Glutathione Assay Kit Liver histopathology	* Female ICR mice		[44]
	Kaempferol	Flavonol	Kaempferol-3-O-glucoside p-coumaric acid kaempferol 3-(4 hydroxyphenyl) propionic acid 3-phenylpropionic acid	Diabetes	* Kaempferol treatment enhanced cellular viability and antiapoptotic activitites which suggested a cytoprotective role	Cytotoxicity assays Western blot analysis Flow cytometry	* Female Sprague-Dawley rats	* RIN-5F cells	[78]

**Table 2:** Representative Flavonoids Mechanisms and their Underlying Anticancer Effects.

Targeted Pathway	Name of Flavonoid	Flavonoid Subclass	Metabolites Produced by Gut Microbiota	Disease(s)	Influence of Flavonoids on Disease	Methods of Testing	Model Used		References
							In vivo	In vitro	
Enzymatic Modification	Lycopene	Carotenoid	No available data	Cancer	* Lycopene administration could suppress gastric cancer through the reduction of lipid peroxidation and the enhancement of GSH-dependent enzymes such as glutathione reductase and glutathione-s-transferase	Histopathological examination	* Male Wistar rats		[98]
NF-κB Pathway	Genistein	Isoflavones	Dihydrogenistein 6-hydroxy-O-desmethylangolensin 2-(4-hydroxyphenyl) propionic acid	Cancer	* Genistein treatment significantly inhibited the activities of nuclear factor-kappa B (NF-κB) signaling pathway which resulted in a reduced COX-2 protein level	ELISA Flow cytometry Western blot analysis Morphological determination		* Human gastric cancer BGC-823 cells	[69]
	Morin	Flavonol	Morin glucuronides Morin sulfates	Cancer	* Morin inhibited the activation of nuclear factor-kappa B (NF-κB) pathway, thus reducing the production of IL-6 and IL-8	Reverse transcription PCR Apoptosis assay ELISA Western blot analysis Cell viability Immunofluorescent staining assay	* Athymic nude mice	* HT-29 cells * HCT-116 cells	[68]
	Chrysin	Flavones	No available data	Cancer	* Chrysin downregulated the activities of nuclear factor-kappa B (NF-κB) which reduced the expression of recepteur d’origine nantais (RON), a c-Met factor receptor critical for gastric cancer invasion and metastasis	Reverse transcription PCR ELISA Western blot analysis Cell viability		* AGS human gastric cancer cells	[67]
	Quercetin	Flavonol	3,4-dihydroxyphenylacetic acid 3-(3-hydroxyphenyl) propionic acid 3,4-dihydroxybenzoic 4-hydroxybenzoic acid	Cancer	* Administration of quercetin showed an antimetastatic effect against gastric cancer through the inhibition of nuclear factor-kappa B (NF-κB)	uPA activity assay Western blot analysis Cell viability		* MGC803 cells * GC7901 cells * AGS human gastric cancer cells	[70]
AMPK Pathway	Genistein	Isoflavones	Dihydrogenistein 6-hydroxy-O-desmethylangolensin 2-(4-hydroxyphenyl) propionic acid	Cancer	* Combination of 5-Fluorouracil (5-FU) and genistein in colon cancer cells activated AMPK activities and upregulated p53, p21, and Bax signals	Reverse transcription PCR ELISA Western blot analysis Cell viability		* HT-29 cells	[99]
Apoptosis	Morin	Flavonol	Morin glucuronides Morin sulfates	Cancer	* The administration of morin induced apoptosis through the increased level of ROS	Electron microscopy Flow cytometry Cell viability assays Immunoblotting Quantitative PCR Flow cytometry		* SW480 cells	[53]
	Fisetin	Flavonol	No available data	Cancer	* Fisetin treatment significantly induced the expression of pro-apoptotic Caspase-9 and Caspase-3 and inhibited the anti-apoptotic Bcl-2	Histopathological analysis ELISA Estimation of lipid peroxidation	* Male Wistar rats		[100]
	Hesperidin	Flavanones	Hesperetin	Cancer	* Cells treated with hesperidin showed a decreased expression of B-cell CLL/lymphoma 2 (BCL2) mRNA, and an increased expression of BCL2-associated X protein (BAX). * Hesperidin induced the expression of pro-apoptotic Caspase-3	Reverse transcription PCR Caspase3 activity assay Western blot analysis Cell viability		* SNU-C4 cells	[101]
	Kaempferol	Flavonol	Kaempferol-3-O-glucoside p-coumaric acid kaempferol 3-(4 hydroxyphenyl) propionic acid 3-phenylpropionic acid	Cancer	* Kaempferol treatment increased the number of early apoptotic cells * Treatment with Kaempferol increased the levels of cleaved caspase-9, caspase-3, and caspase-7	LDH assays Cell viability Immunoprecipitation (IP) assay Reverse transcription PCR Western blot analysis Caspase-8 assays		* AGS cells * SNU-216 cells * NCI-N87 cells * HT-29 cells	[51,102]
	Apigenin	Flavones	Apigenin 3-(4-hydroxyphenyl) propionic acid	Cancer	* Apigenin treatment reduced cellular proliferation and induced apoptosis through the suppression of the antiapoptotic proteins Bcl-xL and Mcl-1 * Apigenin reported to induce apoptosis through the inhibition of STAT3 phosphorylation	Immunohistochemistry Cell proliferation assay Western blot analysis		* HT29 cells	[49]
	Genistein	Isoflavones	Dihydrogenistein 6-hydroxy-O-desmethylangolensin 2-(4-hydroxyphenyl) propionic acid	Cancer	* Genistein induced apoptosis by promoting the expression of Bax/Bcl-2 and caspase-3	Quantitative PCR Flow cytometry Immunofluorescence imaging Protein extraction Immunoblot		* HT-29 cells	[50]
	Chrysin	Flavones	No available data	Cancer	* Chrysin reduced cellular proliferation and induced apoptosis in colon cancer cells through caspase-3 and caspase-9	Caspase-3 and Caspase-9 Asctivity Morphological Assessments Reverse transcription PCR Flow cytometry	* Male BALB/c mice	* CT26 cells	[52]
	Tangeretin	Flavones	Tangeretin-O-glucuronides	Cancer	* Tangeretin administration induced apoptosis by upregulating the activities of caspase 3, 8, and 9 * Tangeretin treatment decreased the membrane potential of mitochondria (MMP)	Caspase-3 and Caspase-9 Asctivity Western blot analysis MTT assay for cell viability Flow cytometry		* AGS cells	[103]

## Abbreviations

GI	Gastrointestinal
CRC	Colorectal cancer
GC	Gastric cancer
IBD	Inflammatory bowel disease
SCFA	Short chain fatty acid
TLR	Toll-like receptor
LPS	Lipopolysaccharide
GLUT	Glucose transporter
AMPK	AMP-activated protein kinase
NF-κB	Nuclear factor kappa
ROS	Reactive oxygen species
EMT	Epithelial-mesenchymal transition
FISH	Fluorescence in situ hybridization
COX-2	Cyclooxygenase-2
G6Pase	Glucose 6-phosphatase
PEPCK	Phosphoenolpyruvate carboxykinase
PCR	Polymerase chain reaction
IP	Immunoprecipitation assay

## References

[1] Patel D.K., Kumar R., Laloo D., Hemalatha S. (2012). Diabetes mellitus: An overview on its pharmacological aspects and reported medicinal plants having antidiabetic activity. Asian Pac. J. Trop. Biomed..

[2] Banday M.Z., Sameer A.S., Nissar S. (2020). Pathophysiology of diabetes: An overview. Avicenna J. Med..

[3] Kharroubi A.T., Darwish H.M. (2015). Diabetes mellitus: The epidemic of the century. World J. Diabetes.

[4] Chen L., Magliano D.J., Zimmet P.Z. (2011). The worldwide epidemiology of type 2 diabetes mellitus-present and future perspectives. Nat. Rev. Endocrinol..

[5] Reyes J., Tripp-Reimer T., Parker E., Muller B., Laroche H. (2017). Factors Influencing Diabetes Self-Management Among Medically Underserved Patients with Type II Diabetes. Glob. Qual. Nurs. Res..

[6] Chawla A., Chawla R., Jaggi S. (2016). Microvasular and macrovascular complications in diabetes mellitus: Distinct or continuum?. Indian J. Endocrinol. Metab..

[7] Lin X., Xu Y., Pan X., Xu J., Ding Y., Sun X., Shan P.F. (2020). Global, regional, and national burden and trend of diabetes in 195 countries and territories: An analysis from 1990 to 2025. Sci. Rep..

[8] Ramachandran A. (2014). Know the signs and symptoms of diabetes. Indian J. Med. Res..

[9] Dhatariya K. (2019). Diabetes: The place of new therapies. Adv. Endocrinol. Metab..

[10] Blaslov K., Naranda F.S., Kruljac I., Renar I.P. (2018). Treatment approach to type 2 diabetes: Past, present and future. World J. Diabetes.

[11] Hassanzade J., Molavi E.V.H., Farahmand M., Rajaiifard A.R. (2011). Incidence and Mortality Rate of Common Gastrointestinal Cancers in South of Iran, a Population Based Study. Iran. J. Cancer Prev..

[12] Bordry N., Astaras C., Ongaro M., Goossens N., Frossard J.L., Koessler T. (2021). Recent advances in gastrointestinal cancers. World J. Gastroenterol..

[13] Abotaleb M., Samuel S.M., Varghese E., Varghese S., Kubatka P., Liskova A., Busselberg D. (2018). Flavonoids in Cancer and Apoptosis. Cancers.

[14] Zali H., Rezaei-Tavirani M., Azodi M. (2011). Gastric cancer: Prevention, risk factors and treatment. Gastroenterol. Hepatol. Bed Bench..

[15] Han C.J., Reding K., Cooper B.A., Paul S.M., Conley Y.P., Hammer M., Miaskowski C. (2019). Symptom Clusters in PatientsWith Gastrointestinal Cancers Using Different Dimensions of the Symptom Experience. J. Pain Symptom Manag..

[16] Machlowska J., Baj J., Sitarz M., Maciejewski R., Sitarz R. (2020). Gastric Cancer: Epidemiology, Risk Factors, Classification, Genomic Characteristics and Treatment Strategies. Int. J. Mol. Sci..

[17] Correa P. (2020). Gastric cancer: Overview. Gastroenterol. Clin. N. Am..

[18] Matsuoka T., Yashiro M. (2020). Precision medicine for gastrointestinal cancer: Recent progress and future perspective. World J. Gastrointest Oncol..

[19] Panche A.N., Diwan A.D., Chandra S.R. (2016). Flavonoids: An overview. J. Nutr. Sci..

[20] Kozlowska A., Szostak-Wegierek D. (2014). Flavonoids—food sources and health benefits. Rocz. Panstw. Zakl. Hig..

[21] Ullah A., Munir S., Badshah S.L., Khan N., Ghani L., Poulson B.G., Jaremko M. (2020). Important Flavonoids and Their Role as a Therapeutic Agent. Molecules.

[22] Juca M.M., Cysne Filho F.M.S., de Almeida J.C., Mesquita D.D.S., Barriga J.R.M., Dias K.C.F., Vasconcelos S.M.M. (2020). Flavonoids: Biological activities and therapeutic potential. Nat. Prod. Res..

[23] Sok Yen F., Shu Qin C., Tan Shi Xuan S., Jia Ying P., Yi Le H., Darmarajan T., Salvamani S. (2021). Hypoglycemic Effects of Plant Flavonoids: A Review. Evid Based Complement. Altern. Med..

[24] Testa R., Bonfigli A.R., Genovese S., De Nigris V., Ceriello A. (2016). The Possible Role of Flavonoids in the Prevention of Diabetic Complications. Nutrients.

[25] Rodriguez-Garcia C., Sanchez-Quesada C., Gaforio J.J. (2019). Dietary Flavonoids as Cancer Chemopreventive Agents: An Updated Review of Human Studies. Antioxidants.

[26] Vitelli Storelli F., Molina A.J., Zamora-Ros R., Fernandez-Villa T., Roussou V., Romaguera D., Martin V. (2019). Flavonoids and the Risk of Gastric Cancer: An Exploratory Case-Control Study in the MCC-Spain Study. Nutrients.

[27] Bull M.J., Plummer N.T. (2014). Part 1: The Human Gut Microbiome in Health and Disease. Integr. Med. (Encinitas).

[28] Thursby E., Juge N. (2017). Introduction to the human gut microbiota. Biochem. J..

[29] Al-Ishaq R.K., Liskova A., Kubatka P., Busselberg D. (2021). Enzymatic Metabolism of Flavonoids by Gut Microbiota and Its Impact on Gastrointestinal Cancer. Cancers.

[30] Forslund K., Hildebrand F., Nielsen T., Falony G., Le Chatelier E., Sunagawa S., Pedersen O. (2015). Disentangling type 2 diabetes and metformin treatment signatures in the human gut microbiota. Nature.

[31] Glassner K.L., Abraham B.P., Quigley E.M.M. (2020). The microbiome and inflammatory bowel disease. J. Allergy Clin. Immunol..

[32] Chen Z., Radjabzadeh D., Chen L., Kurilshikov A., Kavousi M., Ahmadizar F., Voortman T. (2021). Association of Insulin Resistance and Type 2 Diabetes With Gut Microbial Diversity: A Microbiome-Wide Analysis From Population Studies. JAMA Netw. Open.

[33] Cheng W.Y., Wu C.Y., Yu J. (2020). The role of gut microbiota in cancer treatment: Friend or foe?. Gut.

[34] Al-Ishaq R.K., Abotaleb M., Kubatka P., Kajo K., Busselberg D. (2019). Flavonoids and Their Antidiabetic Effects: Cellular Mechanisms and Effects to Improve Blood Sugar Levels. Biomolecules.

[35] Al-Ishaq R.K., Overy A.J., Busselberg D. (2020). Phytochemicals and Gastrointestinal Cancer: Cellular Mechanisms and Effects to Change Cancer Progression. Biomolecules.

[36] D’Arcy M.S. (2019). Cell death: A review of the major forms of apoptosis, necrosis and autophagy. Cell Biol. Int..

[37] Aachoui Y., Sagulenko V., Miao E.A., Stacey K.J. (2013). Inflammasome-mediated pyroptotic and apoptotic cell death, and defense against infection. Curr. Opin. Microbiol..

[38] Singh R., Letai A., Sarosiek K. (2019). Regulation of apoptosis in health and disease: The balancing act of BCL-2 family proteins. Nat. Rev. Mol. Cell Biol..

[39] Slika H., Mansour H., Wehbe N., Nasser S.A., Iratni R., Nasrallah G., Eid A.H. (2022). Therapeutic potential of flavonoids in cancer: ROS-mediated mechanisms. Biomed. Pharm..

[40] Naudi A., Jove M., Ayala V., Cassanye A., Serrano J., Gonzalo H., Pamplona R. (2012). Cellular dysfunction in diabetes as maladaptive response to mitochondrial oxidative stress. Exp. Diabetes Res..

[41] Pitocco D., Tesauro M., Alessandro R., Ghirlanda G., Cardillo C. (2013). Oxidative stress in diabetes: Implications for vascular and other complications. Int. J. Mol. Sci..

[42] Ma P., Mao X.Y., Li X.L., Ma Y., Qiao Y.D., Liu Z.Q., Cao Y.G. (2015). Baicalin alleviates diabetes associated cognitive deficits via modulation of mitogen-activated protein kinase signaling, brain derived neurotrophic factor and apoptosis. Mol. Med. Rep..

[43] Dong Y., Xing Y., Sun J., Sun W., Xu Y., Quan C. (2020). Baicalein Alleviates Liver Oxidative Stress and Apoptosis Induced by High-Level Glucose through the Activation of the PERK/Nrf2 Signaling Pathway. Molecules.

[44] Parmar M.S., Syed I., Gray J.P., Ray S.D. (2015). Curcumin, Hesperidin, and Rutin Selectively Interfere with Apoptosis Signaling and Attenuate Streptozotocin-Induced Oxidative Stress-Mediated Hyperglycemia. Curr. Neurovasc. Res..

[45] Tian M., Han Y.B., Zhao C.C., Liu L., Zhang F.L. (2021). Hesperidin alleviates insulin resistance by improving HG-induced oxidative stress and mitochondrial dysfunction by restoring miR-149. Diabetol. Metab. Syndr..

[46] Ghorbani A. (2017). Mechanisms of antidiabetic effects of flavonoid rutin. Biomed. Pharm..

[47] Wang J., Wang R., Li J., Yao Z. (2021). Rutin alleviates cardiomyocyte injury induced by high glucose through inhibiting apoptosis and endoplasmic reticulum stress. Exp. Med..

[48] Pfeffer C.M., Singh A.T.K. (2018). Apoptosis: A Target for Anticancer Therapy. Int. J. Mol. Sci..

[49] Maeda Y., Takahashi H., Nakai N., Yanagita T., Ando N., Okubo T., Takiguchi S. (2018). Apigenin induces apoptosis by suppressing Bcl-xl and Mcl-1 simultaneously via signal transducer and activator of transcription 3 signaling in colon cancer. Int. J. Oncol..

[50] Zhou P., Wang C., Hu Z., Chen W., Qi W., Li A. (2017). Genistein induces apoptosis of colon cancer cells by reversal of epithelial-to-mesenchymal via a Notch1/NF-kappaB/slug/E-cadherin pathway. BMC Cancer.

[51] Kim T.W., Lee S.Y., Kim M., Cheon C., Ko S.G. (2018). Kaempferol induces autophagic cell death via IRE1-JNK-CHOP pathway and inhibition of G9a in gastric cancer cells. Cell Death Dis..

[52] Bahadori M., Baharara J., Amini E. (2016). Anticancer Properties of Chrysin on Colon Cancer Cells, In vitro and In vivo with Modulation of Caspase-3, -9, Bax and Sall4. Iran. J. Biotechnol..

[53] Sithara T., Arun K.B., Syama H.P., Reshmitha T.R., Nisha P. (2017). Morin Inhibits Proliferation of SW480 Colorectal Cancer Cells by Inducing Apoptosis Mediated by Reactive Oxygen Species Formation and Uncoupling of Warburg Effect. Front. Pharm..

[54] Zhang J., Wu D., Vikash S.J., Wang J., Yi J., Dong W. (2015). Hesperetin Induces the Apoptosis of Gastric Cancer Cells via Activating Mitochondrial Pathway by Increasing Reactive Oxygen Species. Dig. Dis Sci..

[55] Yang C., Song J., Hwang S., Choi J., Song G., Lim W. (2021). Apigenin enhances apoptosis induction by 5-fluorouracil through regulation of thymidylate synthase in colorectal cancer cells. Redox Biol..

[56] Napetschnig J., Wu H. (2013). Molecular basis of NF-kappaB signaling. Annu. Rev. Biophys..

[57] Park M.H., Hong J.T. (2016). Roles of NF-kappaB in Cancer and Inflammatory Diseases and Their Therapeutic Approaches. Cells.

[58] Paneni F., Beckman J.A., Creager M.A., Cosentino F. (2013). Diabetes and vascular disease: Pathophysiology, clinical consequences, and medical therapy: Part I. Eur. Heart J..

[59] Suryavanshi S.V., Kulkarni Y.A. (2017). NF-kappabeta: A Potential Target in the Management of Vascular Complications of Diabetes. Front. Pharm..

[60] Chaithongyot S., Jantaree P., Sokolova O., Naumann M. (2021). NF-kappaB in Gastric Cancer Development and Therapy. Biomedicines.

[61] Li G., Ding K., Qiao Y., Zhang L., Zheng L., Pan T., Zhang L. (2020). Flavonoids Regulate Inflammation and Oxidative Stress in Cancer. Molecules.

[62] Jubaidi F.F., Zainalabidin S., Taib I.S., Hamid Z.A., Budin S.B. (2021). The Potential Role of Flavonoids in Ameliorating Diabetic Cardiomyopathy via Alleviation of Cardiac Oxidative Stress, Inflammation and Apoptosis. Int. J. Mol. Sci..

[63] Yin H., Huang L., Ouyang T., Chen L. (2018). Baicalein improves liver inflammation in diabetic db/db mice by regulating HMGB1/TLR4/NF-kappaB signaling pathway. Int. Immunopharmacol..

[64] Malik S., Suchal K., Khan S.I., Bhatia J., Kishore K., Dinda A.K., Arya D.S. (2017). Apigenin ameliorates streptozotocin-induced diabetic nephropathy in rats via MAPK-NF-kappaB-TNF-alpha and TGF-beta1-MAPK-fibronectin pathways. Am. J. Physiol. Ren. Physiol..

[65] Li L., Luo W., Qian Y., Zhu W., Qian J., Li J., Liang G. (2019). Luteolin protects against diabetic cardiomyopathy by inhibiting NF-kappaB-mediated inflammation and activating the Nrf2-mediated antioxidant responses. Phytomedicine.

[66] Mahmoud M.F., Hassan N.A., El Bassossy H.M., Fahmy A. (2013). Quercetin protects against diabetes-induced exaggerated vasoconstriction in rats: Effect on low grade inflammation. PLoS ONE.

[67] Xia Y., Lian S., Khoi P.N., Yoon H.J., Han J.Y., Chay K.O., Jung Y.D. (2015). Chrysin inhibits cell invasion by inhibition of Recepteur d’origine Nantais via suppressing early growth response-1 and NF-kappaB transcription factor activities in gastric cancer cells. Int. J. Oncol..

[68] Chen R., Zhang L. (2019). Morin inhibits colorectal tumor growth through inhibition of NF-kappaB signaling pathway. Immunopharmacol. Immunotoxicol..

[69] Li Y.S., Wu L.P., Li K.H., Liu Y.P., Xiang R., Zhang S.B., Zhang L.Y. (2011). Involvement of nuclear factor kappaB (NF-kappaB) in the downregulation of cyclooxygenase-2 (COX-2) by genistein in gastric cancer cells. J. Int. Med. Res..

[70] Li H., Chen C. (2018). Quercetin Has Antimetastatic Effects on Gastric Cancer Cells via the Interruption of uPA/uPAR Function by Modulating NF-kappab, PKC-delta, ERK1/2, and AMPKalpha. Integr. Cancer.

[71] Mihaylova M.M., Shaw R.J. (2011). The AMPK signalling pathway coordinates cell growth, autophagy and metabolism. Nat. Cell Biol..

[72] Hardie D.G., Carling D., Gamblin S.J. (2011). AMP-activated protein kinase: Also regulated by ADP?. Trends Biochem. Sci..

[73] Wang S., Song P., Zou M.H. (2012). AMP-activated protein kinase, stress responses and cardiovascular diseases. Clin. Sci..

[74] Coughlan K.A., Valentine R.J., Ruderman N.B., Saha A.K. (2014). AMPK activation: A therapeutic target for type 2 diabetes?. Diabetes Metab. Syndr. Obes..

[75] Li W., Saud S.M., Young M.R., Chen G., Hua B. (2015). Targeting AMPK for cancer prevention and treatment. Oncotarget.

[76] Dhanya R., Arya A.D., Nisha P., Jayamurthy P. (2017). Quercetin, a Lead Compound against Type 2 Diabetes Ameliorates Glucose Uptake via AMPK Pathway in Skeletal Muscle Cell Line. Front. Pharm..

[77] Cheong S.H., Furuhashi K., Ito K., Nagaoka M., Yonezawa T., Miura Y., Yagasaki K. (2014). Daidzein promotes glucose uptake through glucose transporter 4 translocation to plasma membrane in L6 myocytes and improves glucose homeostasis in Type 2 diabetic model mice. J. Nutr. Biochem..

[78] Varshney R., Gupta S., Roy P. (2017). Cytoprotective effect of Kaempferol against palmitic acid-induced pancreatic beta-cell death through modulation of autophagy via AMPK/mTOR signaling pathway. Mol. Cell Endocrinol..

[79] Talib W.H., Awajan D., Hamed R.A., Azzam A.O., Mahmod A.I., Al-Yasari I.H. (2022). Combination Anticancer Therapies Using Selected Phytochemicals. Molecules.

[80] Zallot R., Oberg N., Gerlt J.A. (2021). Discovery of new enzymatic functions and metabolic pathways using genomic enzymology web tools. Curr. Opin. Biotechnol..

[81] Robinson P.K. (2015). Enzymes: Principles and biotechnological applications. Essays Biochem..

[82] Asmat U., Abad K., Ismail K. (2016). Diabetes mellitus and oxidative stress-A concise review. Saudi Pharm. J..

[83] Bansal A., Simon M.C. (2018). Glutathione metabolism in cancer progression and treatment resistance. J. Cell Biol..

[84] Rabbani N., Thornalley P.J. (2019). Hexokinase-2 Glycolytic Overload in Diabetes and Ischemia-Reperfusion Injury. Trends Endocrinol. Metab..

[85] Choi M.S., Jung U.J., Yeo J., Kim M.J., Lee M.K. (2008). Genistein and daidzein prevent diabetes onset by elevating insulin level and altering hepatic gluconeogenic and lipogenic enzyme activities in non-obese diabetic (NOD) mice. Diabetes Metab. Res. Rev..

[86] Pari L., Srinivasan S. (2010). Antihyperglycemic effect of diosmin on hepatic key enzymes of carbohydrate metabolism in streptozotocin-nicotinamide-induced diabetic rats. Biomed. Pharm..

[87] Patel S.S., Shah R.S., Goyal R.K. (2009). Antihyperglycemic, antihyperlipidemic and antioxidant effects of Dihar, a polyherbal ayurvedic formulation in streptozotocin induced diabetic rats. Indian J. Exp. Biol..

[88] Vanitha P., Uma C., Suganya N., Bhakkiyalakshmi E., Suriyanarayanan S., Gunasekaran P., Ramkumar K.M. (2014). Modulatory effects of morin on hyperglycemia by attenuating the hepatic key enzymes of carbohydrate metabolism and beta-cell function in streptozotocin-induced diabetic rats. Environ. Toxicol. Pharm..

[89] Hanschmann E.M., Godoy J.R., Berndt C., Hudemann C., Lillig C.H. (2013). Thioredoxins, glutaredoxins, and peroxiredoxins--molecular mechanisms and health significance: From cofactors to antioxidants to redox signaling. Antioxid Redox Signal..

[90] Kennedy L., Sandhu J.K., Harper M.E., Cuperlovic-Culf M. (2020). Role of Glutathione in Cancer: From Mechanisms to Therapies. Biomolecules.

[91] Marzocco S., Singla R.K., Capasso A. (2021). Multifaceted Effects of Lycopene: A Boulevard to the Multitarget-Based Treatment for Cancer. Molecules.

[92] JZ A.L., BinMowyna M.N., AlFaris N.A., Alagal R.I., El-Kott A.F., Al-Farga A.M. (2021). Fisetin protects against streptozotocin-induced diabetic cardiomyopathy in rats by suppressing fatty acid oxidation and inhibiting protein kinase R. Saudi Pharm. J..

[93] Akiyama S., Katsumata S., Suzuki K., Ishimi Y., Wu J., Uehara M. (2010). Dietary hesperidin exerts hypoglycemic and hypolipidemic effects in streptozotocin-induced marginal type 1 diabetic rats. J. Clin. Biochem. Nutr..

[94] Li H.T., Wu X.D., Davey A.K., Wang J. (2011). Antihyperglycemic effects of baicalin on streptozotocin-nicotinamide induced diabetic rats. Phytother. Res..

[95] Zheng Z.C., Zhu W., Lei L., Liu X.Q., Wu Y.G. (2020). Wogonin Ameliorates Renal Inflammation and Fibrosis by Inhibiting NF-kappaB and TGF-beta1/Smad3 Signaling Pathways in Diabetic Nephropathy. Drug Des. Devel..

[96] Zhang F., Feng J., Zhang J., Kang X., Qian D. (2020). Quercetin modulates AMPK/SIRT1/NF-kappaB signaling to inhibit inflammatory/oxidative stress responses in diabetic high fat diet-induced atherosclerosis in the rat carotid artery. Exp. Med..

[97] Cheong S.H., Furuhashi K., Ito K., Nagaoka M., Yonezawa T., Miura Y., Yagasaki K. (2014). Antihyperglycemic effect of equol, a daidzein derivative, in cultured L6 myocytes and ob/ob mice. Mol. Nutr. Food Res..

[98] Velmurugan B., Bhuvaneswari V., Nagini S. (2002). Antiperoxidative effects of lycopene during N-methyl-N′-nitro-N-nitrosoguanidine-induced gastric carcinogenesis. Fitoterapia.

[99] Hwang J.T., Ha J., Park O.J. (2005). Combination of 5-fluorouracil and genistein induces apoptosis synergistically in chemo-resistant cancer cells through the modulation of AMPK and COX-2 signaling pathways. Biochem. Biophys. Res. Commun.

[100] Fan Q., Wang X., Chinnathambi A., Alharbi S., Wang Q. (2020). Fisetin suppresses 1,2-dimethylhydrazine-induced colon tumorigenesis in Wistar rats. J. King Saud Univ. Sci..

[101] Park H.J., Kim M.J., Ha E., Chung J.H. (2008). Apoptotic effect of hesperidin through caspase3 activation in human colon cancer cells, SNU-C4. Phytomedicine.

[102] Lee H.S., Cho H.J., Yu R., Lee K.W., Chun H.S., Park J.H. (2014). Mechanisms underlying apoptosis-inducing effects of Kaempferol in HT-29 human colon cancer cells. Int. J. Mol. Sci..

[103] Dong Y., Cao A., Shi J., Yin P., Wang L., Ji G., Wu D. (2014). Tangeretin, a citrus polymethoxyflavonoid, induces apoptosis of human gastric cancer AGS cells through extrinsic and intrinsic signaling pathways. Oncol. Rep..

[104] Hanchang W., Khamchan A., Wongmanee N., Seedadee C. (2019). Hesperidin ameliorates pancreatic beta-cell dysfunction and apoptosis in streptozotocin-induced diabetic rat model. Life Sci..

[105] Riahi-Chebbi I., Souid S., Othman H., Haoues M., Karoui H., Morel A., Essafi-Benkhadir K. (2019). The Phenolic compound Kaempferol overcomes 5-fluorouracil resistance in human resistant LS174 colon cancer cells. Sci. Rep..

[106] Zhang Y., Liu D. (2011). Flavonol kaempferol improves chronic hyperglycemia-impaired pancreatic beta-cell viability and insulin secretory function. Eur. J. Pharm..

[107] Zhang X.A., Zhang S., Yin Q., Zhang J. (2015). Quercetin induces human colon cancer cells apoptosis by inhibiting the nuclear factor-kappa B Pathway. Pharm. Mag..

[108] Wang L., Gao M., Kang G., Huang H. (2021). The Potential Role of Phytonutrients Flavonoids Influencing Gut Microbiota in the Prophylaxis and Treatment of Inflammatory Bowel Disease. Front. Nutr..

[109] Pei R., Liu X., Bolling B. (2020). Flavonoids and gut health. Curr. Opin. Biotechnol..

[110] Murota K., Nakamura Y., Uehara M. (2018). Flavonoid metabolism: The interaction of metabolites and gut microbiota. Biosci. Biotechnol. Biochem..

[111] Tilg H., Moschen A.R. (2014). Microbiota and diabetes: An evolving relationship. Gut.

[112] Li W.Z., Stirling K., Yang J.J., Zhang L. (2020). Gut microbiota and diabetes: From correlation to causality and mechanism. World J. Diabetes.

[113] Karlsson F.H., Tremaroli V., Nookaew I. (2013). Gut metagenome in European women with normal, impaired and diabetic glucose control. Nature.

[114] Kim J., Lee H.K. (2021). Potential Role of the Gut Microbiome In Colorectal Cancer Progression. Front. Immunol..

[115] Liu Y., Baba Y., Ishimoto T., Gu X., Zhang J., Nomoto D., Qiu P. (2022). Gut microbiome in gastrointestinal cancer: A friend or foe?. Int. J. Biol Sci..

[116] Tremmel M., Paetz C., Heilmann J. (2021). In Vitro Liver Metabolism of Six Flavonoid C-Glycosides. Molecules.

[117] Wang L., Huang G., Hou R., Qi D., Wu Q., Nie Y., Wei F. (2021). Multi-omics reveals the positive leverage of plant secondary metabolites on the gut microbiota in a non-model mammal. Microbiome.

[118] Sampson L., Rimm E., Hollman P.C., de Vries J.H., Katan M.B. (2002). Flavonol and flavone intakes in US health professionals. J. Am. Diet. Assoc..

[119] Boker K.L., Van der Schouw Y.T., De Kleijn M.J., Jacques P.F., Grobbee D.E., Peeters P.H. (2002). Intake of Dietary Phytoestrogens by Dutch Women. J. Nutr..

[120] Arts I.C., Hollman P.C., Feskens E.J., Bueno de Mesquita H.B., Kromhout D. (2001). Catechin intake and associated dietary and lifestyle factors in a representative sample of Dutch men and women. Eur. J. Clin. Nutr..

[121] Rodriguez De Luna S.L., Ramirez-Garza R.E., Serna Saldivar S.O. (2020). Environmentally Friendly Methods for Flavonoid Extraction from Plant Material: Impact of Their Operating Conditions on Yield and Antioxidant Properties. Sci. World J..

[122] Thilakarathna S.H., Rupasinghe H.P. (2013). Flavonoid bioavailability and attempts for bioavailability enhancement. Nutrients.

[123] Roderburg C., Loosen S.H., Hoyer L., Luedde T., Kostev K. (2022). Prevalence of diabetes mellitus among 80,193 gastrointestinal cancer patients in five European and three Asian countries. J. Cancer Res. Clin. Oncol..

[124] Tseng C.H. (2021). The Relationship between Diabetes Mellitus and Gastric Cancer and the Potential Benefits of Metformin: An Extensive Review of the Literature. Biomolecules.

[125] Tseng C.H., Tseng F.H. (2014). Diabetes and gastric cancer: The potential links. World J. Gastroenterol..

[126] Samec M., Liskova A., Koklesova L., Mersakova S., Strnadel J., Kajo K., Kubatka P. (2021). Flavonoids Targeting HIF-1: Implications on Cancer Metabolism. Cancers.

[127] Kubatka P., Mazurakova A., Samec M., Koklesova L., Zhai K., Al-Ishaq R., Golubnitschaja O. (2021). Flavonoids against non-physiologic inflammation attributed to cancer initiation, development, and progression-3PM pathways. EPMA J..

[128] Zhang S., Jin S., Zhang S., Li Y.Y., Wang H., Chen Y., Lu H. (2022). Vitexin protects against high glucose-induced endothelial cell apoptosis and oxidative stress via Wnt/beta-catenin and Nrf2 signalling pathway. Arch. Physiol. Biochem..

[129] Ling J.Y., Wang Q.L., Liang H.N., Liu Q.B., Yin D.H., Lin L. (2022). Flavonoid-Rich Extract of *Oldenlandia diffusa* (Willd.) Roxb. Inhibits Gastric Cancer by Activation of Caspase-Dependent Mitochondrial Apoptosis. Chin. J. Integr. Med..

